# Leveraging existing 16S rRNA gene surveys to decipher microbial signatures and dysbiosis in cervical carcinogenesis

**DOI:** 10.1038/s41598-024-62531-z

**Published:** 2024-05-21

**Authors:** Xiaoxiao Li, Fenfen Xiang, Tong Liu, Zixi Chen, Mengzhe Zhang, Jinpeng Li, Xiangdong Kang, Rong Wu

**Affiliations:** 1 Department of Laboratory Medicine, Putuo Hospital, Shanghai University of Traditional Chinese Medicine, 164 Lanxi Road, Shanghai, 200062 China; 2 Department of Molecular Science, Uppsala Biocenter, Swedish University of Agricultural Science, Uppsala, Sweden

**Keywords:** Microbiota, HPV, Cervical cancer, 16S rRNA sequencing, Biomarkers, Cancer microenvironment, Cancer, Cervical cancer, Pathogenesis, Bacteria, Clinical microbiology, Microbial communities, Pathogens

## Abstract

The presence of dysbiotic cervicovaginal microbiota has been observed to be linked to the persistent development of cervical carcinogenesis mediated by the human papillomavirus (HPV). Nevertheless, the characteristics of the cervical microbiome in individuals diagnosed with cervical cancer (CC) are still not well understood. Comprehensive analysis was conducted by re-analyzing the cervical 16S rRNA sequencing datasets of a total of 507 samples from six previously published studies. We observed significant alpha and beta diversity differences in between CC, cervical intraepithelial neoplasia (CIN) and normal controls (NC), but not between HPV and NC in the combined dataset. Meta-analysis revealed that opportunistic pernicious microbes *Streptococcus*, *Fusobacterium*, *Pseudomonas* and *Anaerococcus* were enriched in CC, while *Lactobacillus* was depleted compared to NC. Members of *Gardnerella*, *Sneathia*, *Pseudomonas*, and *Fannyhessea* have significantly increased relative abundance compared to other bacteria in the CIN group. Five newly identified bacterial genera were found to differentiate CC from NC, with an area under the curve (AUC) of 0.8947. Moreover, co-occurrence network analysis showed that the most commonly encountered *Lactobacillus* was strongly negatively correlated with *Prevotella*. Overall, our study identified a set of potential biomarkers for CC from samples across different geographic regions. Our meta-analysis provided significant insights into the characteristics of dysbiotic cervicovaginal microbiota undergoing CC, which may lead to the development of noninvasive CC diagnostic tools and therapeutic interventions.

## Introduction

CC is the fourth most common malignant tumor in women, but the incidence and mortality rank second place in countries with a low development index^1^. The key etiological factor in the development of CIN and CC is well recognised to be persistent infection with high-risk HPV. Nevertheless, it is widely recognised that HPV alone is insufficient to cause cervical malignant transformation. Numerous factors have been linked to the occurrence of CIN, and it has been suggested that the composition of cervical microbiota has a significant role in the progression of HPV infection, ultimately leading to the formation of CIN or CC^2–4^. The potential impact of this phenomenon on the development of CC lies in its ability to modify various aspects of the host’s inflammatory, genomic, and metabolic processes^5,6^.

An optimal cervicovaginal status was commonly associated with low microbial diversity and prevalence of *Lactobacillus. Lactobacillus* spp., which produce lactic acid and bacteriocin, were the key microbes in healthy women, participating in creating the stability of the cervicovaginal microbial composition and maintaining a low pH environment^7,8^. With the development of next-generation sequencing technology, increasing evidence has shown that cervical microbiota dysbiosis, as an important environmental factor, may lead to bacterial vaginosis (BV)^9^, sexually transmitted infections (STIs)^10^, HPV infection and the subsequent development of cervical lesions^11–16^. There were five community state types (CSTs). CST I, II, III, and V were dominated by *Lactobacillus crispatus* (*L. crispatus*), *Lactobacillus gasseri* (*L. gasseri*), *Lactobacillus iners* (*L. iners*) and *Lactobacillus jessenii* (*L. jessenii*) respectively. CST IV was mainly composed by a high abundance of anaerobic bacteria^17^. Prior research has indicated that *L. crispatus* is more prevalent among women who do not have HPV infection or cancer lesions, but *L. iners* and non-Lactobacillus species are more frequently observed in women with HPV infection and patients who have cancer lesions^18–20^. Numerous research have been conducted to investigate the cervical microbiota and its association with cervical lesions in various populations. However, there have been conflicting findings regarding microbial distinctions between individuals with and without cervical lesions. For example, some taxa, including *Aerococcus*, *Coriobacteria* and *Fannyhessea* were reported to be enriched in CIN^21^. Whereas, Audirac-Chalifour et al.^22^ found that *Sneathia* and *Fusobacterium* were the predominant taxa in CIN and CC, respectively. The key issue related to the differences in cervical microbiota between cervical lesions and health controls is the lack of apparent reproducibility in different studies when identifying the microbiome characteristics of cervical lesions. Besides, the development of non-invasive and sensitive early diagnosis tests for CC and CIN based on cervical microbiota is meaningful.

Variation in study objectives and analytical processing can influence findings. Herein, we systematically reviewed the literature on cervical carcinogenesis microbiota, and used a consistent analytical approach on pooled 16S rRNA gene raw sequences from 6 studies to identify CC-associated microbiota. We sought to determine the differences in alpha-diversity, beta-diversity, microbial compositions and taxonomic alterations across the stages of CC development. Moreover, the bacterial biomarkers for classifications of different disease groups and bacterial taxa correlation were also examined.

## Materials and methods

### Dataset acquisition and study inclusion criteria

A systematic searches for public available data were performed on the NCBI Sequence Read Archive (SRA, http://www.ncbi.nlm.nih.gov/sra), European Nucleotide Archive (ENA, http://www.ebi.ac.uk/ena) and Genome Sequence Archive (GSA, https://ngdc.cncb.ac.cn/gsa/) databases using the search term “cervical cancer”, “HPV”, “cervical intraepithelial neoplasia” and “16S rRNA”, and limiting search results by “Bioproject”. Bioproject accession numbers containing high throughput sequencing reads and associated metadata were collected (Table 1). Then, we retrieved articles from NCBI PubMed or Google Scholar using submitted Bioproject information. The studies incorporated in this meta-analysis were mandated to meet the following criteria: (a) they had to involve samples obtained from the human cervix, (b) they needed to have have sequenced by NGS for 16S rRNA gene, (c) they had non-use of douches in previous days of sampling, (d) not having records of antibiotic or antifungal or antiviral usage within the previous three months of sampling, (e) to have associated metadata, sequencing data and barcodes from the public database or provided by the authors until November 2022 upon request by emails. In order to facilitate comparisons of data across studies, a preformatted metadata file including sample ID, study ID, sequencing type, country and diagnosis was collected from public database or request from authors. Finally, we obtained 16S rRNA sequence data and sufficient metadata (containing information differentiating samples by diagnosis) from 6 studies for subsequent analyses^22–27^. An additional 23 studies related to cervical microbiota of cervical lesions were excluded due to incomplete information on sequences, metadata or barcodes or the same sequences^15,16,21,28–47^. The downloaded datasets were grouped according to the following stages: NC, HPV, CIN and CC.

**Table 1:** Summary of 16S-tag studies on cervicovaginal microbiota-related information.

Data set	Bioproject	Country	*Source	NC	HPV	LSIL	HSIL	CC	Instrument	Region
Cheng	PRJEB34755	Sweden	Author	113	144	0	0	0	MiSeq	V3-V4
Xie	PRJNA595048	China	SRA	25	0	1	18	18	NovaSeq	V4
Audirac-Chalifour	PRJNA308947	Mexico	SRA	7	10	0	4	8	454	V3-V4
Ilhan	PRJNA518153	United States	SRA	18	11	12	27	10	MiSeq	V4
Kang	PRJNA692362	Korea	Author	7	0	0	8	8	PGM	V3
Wei	PRJNA551647	China	SRA	30	30	0	0	0	MiSeq	V3-V4
Mei	PRJCA007388	China	NA	42	28	0	0	0	MiSeq	V3-V4
Chen	PRJNA415526	China	NA	68	78	51	23	9	MiSeq	V3-V4
Onywera	PRJNA473351	South Africa	NA	56	29	0	0	0	MiSeq	V3-V4
Borgogna	PRJNA391039	United States	NA	13	26	0	0	0	454	V1-V3
McKee	PRJNA622998	United States	NA	89	110	45	64	0	MiSeq	V4
Lin	PRJCA007758	China	NA	60	0	60	0	0	MiSeq	V3-V4
Wang	PRJNA815961	China	NA	14	0	9	29	10	MiSeq	V4
Tango	PRJEB5760	Korea	NA	50	0	0	25	17	454	–
Nieves-Ramírez	PRJNA766648	Mexico	NA	107	0	90	31	0	HiSeq	V3
Mitra	PRJEB7756	England	NA	20	0	52	92	5	MiSeq	V1-V2
Bokulich	PRJNA518153	United States	NA	18	9	10	27	8	MiSeq	V4
Chao	PRJCA005712	China	NA	103	86	0	83	0	HiSeq	V4
Piyathilake	PRJNA392354	United States	NA	0	0	340	90	0	MiSeq	V4
Fang	PRJNA846153	China	NA	20	20	0	0	0	NovaSeq	V3-V4
Zhou	PRJNA548879	China	NA	20	42	0	0	0	HiSeq	V3-V4
Godoy-Vitorino	PRJNA429969	United States	NA	10	52	0	0	0	MiSeq	V4
Paola	PRJEB18720	Italy	NA	17	55	0	0	0	454	V3-V5
Lee	PRJEB3342	Korea	NA	45	23	0	0	0	454	V2-V3
Fan	PRJNA725946	China	NA	54	0	0	0	65	HiSeq	V3-V4
Wang2021	PRJNA687331	China	NA	40	0	0	0	26	MiSeq	V3-V4
Lam	PRJNA431248	United States	NA	0	0	0	0	58	MiSeq	V4
Zhang2018	PRJNA319915	China	NA	64	0	62	40	0	HiSeq	V3-V4
Zhang2022	PRJNA787754	China	NA	113	159	47	37	0	NovaSeq	–

*NC* normal control, *HPV* HPV infection, *CIN* cervical intraepithelial neoplasia; Low-grade squamous intraepithelial lesion (women diagnosed with HPV infection and cervical intraepithelial neoplasia 1) [CIN1]; HSIL, high-grade squamous intraepithelial lesion (included CIN2 and CIN3).*CC* cervical cancer.

*NA indicates studies were not included in the analysis due to the datasets without sufficient metadata, without barcode sequences to splits the datasets, or low sequencing quality.

### Sequence processing

The raw data from all datasets were downloaded in the sequence read archive (SRA) and converted into fastq format using SRA Toolkit. Next, all downloaded 16S rRNA gene sequences were processed using the open-source DADA2 for quality control and denoising using parametric error model in R (version 1.16)^48^. Filtering, learning errors, dereplication, amplicon sequence variant (ASV) inference and chimera removal were conducted for each study. Subsequently, taxonomy was assigned using Silva 138 rRNA database using the Naïve Bayesian Classifier algorithm default in DADA2^49^. The merging of paired-end reads was conducted when the overlapping region between the reads exceeded a length of 20 base pairs following the truncation process. Otherwise, only forward reads were used for the following analysis. The within-sample (alpha) diversity metrics including evenness and Shannon diversity were calculated based on the ASV table in each sample in R using vegan package. The between-sample (beta) diversity was assessed based on Bray–Curtis distance. Principal coordinate analysis (PCoA) was used to visualize the distance using PAST (version 3.0) software, and the differences between groups were determined using permutational multivariate analysis of variance (PERMANOVA) with 999 permutations.

### Statistical analysis

Significantly altered taxa among different stage groups were determined by the linear discriminant analysis (LDA) effect size (LEfSe) method with a cutoff LDA score > 3 and *p*-value < 0.05. STAMP software (version 2.1.3) with extend error bar method was used to explore differentially abundant bacteria at genus level between two groups (i.e. NC vs CC), and the significance criteria were *p*-value < 0.05 using two-sided Fishers exact test. Afterward, logistic regression models were built using the selected bacterial genera biomarker with a backward stepwise selection algorithm using leaps package. The receiver operating characteristic (ROC) analysis was used to illustrate the performances of classification models. The statistical analyses were performed using GraphPad Prism software (version 9.3). Pairwise and multiple groups comparisons were performed using the two-sided Wilcoxon rank-sum test and the Kruskal–Wallis test, respectively. Spearman correlation test was used for correlation analyses for the representative (top 15) genera and displayed using R software (version 4.1.2) with corrplot, ggplot2 and ggpubr packages. Bubble plots were made using the ggplot2 and reshape 2 packages, and a heatmap plot was conducted with the pheatmap package in R.

## Results

### Alpha and beta diversity differences

**Figure 1:**
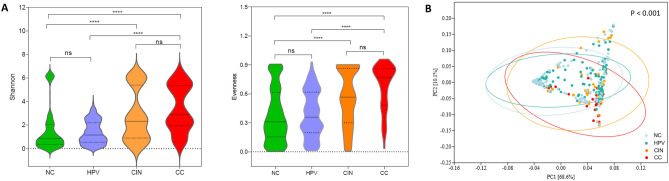
Bacterial diversity for patients in each stage group. (**A**) Alpha-diversity was estimated by Shannon and evenness indexes in each group. The solid black line indicated the corresponding median value in each group. Pairwise comparisons were performed using Wilcoxon rank-sum test. Asterisks mean differences between the two groups are statistically significant (*p* < 0.0001). ns, no significant difference. (**B**) Principal coordinate analysis (PCoA) for all included samples based on Bray–Curtis distance. *p*-value was estimated by permutational multivariate analysis of variance (PERMANOVA).

Quality-filtered 16S rRNA gene sequences from 507 cervical epithelial scrapings samples were available for meta-analysis. Sequences were from six independent studies and five countries (Table 1). To compare the alpha diversity between different disease stages, we observed that the NC group had significantly lower Shannon diversity and evenness when compared CIN and CC groups (*p* < 0.0001) (Fig. 1A). There were no significant differences between NC and HPV groups, CIN and CC groups (*p* > 0.05). Moreover, the microbial diversities exhibited an increasing trend with disease progression (Fig. 1A). We next analyzed whether there were differences in the structures of microbial communities associated with different disease stages. Beta diversity was visualized by principal coordinate analysis (PCoA) based on the Bray–Curtis distance. Overall cervical microbial community structures of four disease stages were significantly different (PERMANOVA, F = 11.54, *p* < 0.001) (Fig. 1B).

### Characteristics of the core cervicovaginal microbiota

**Figure 2:**
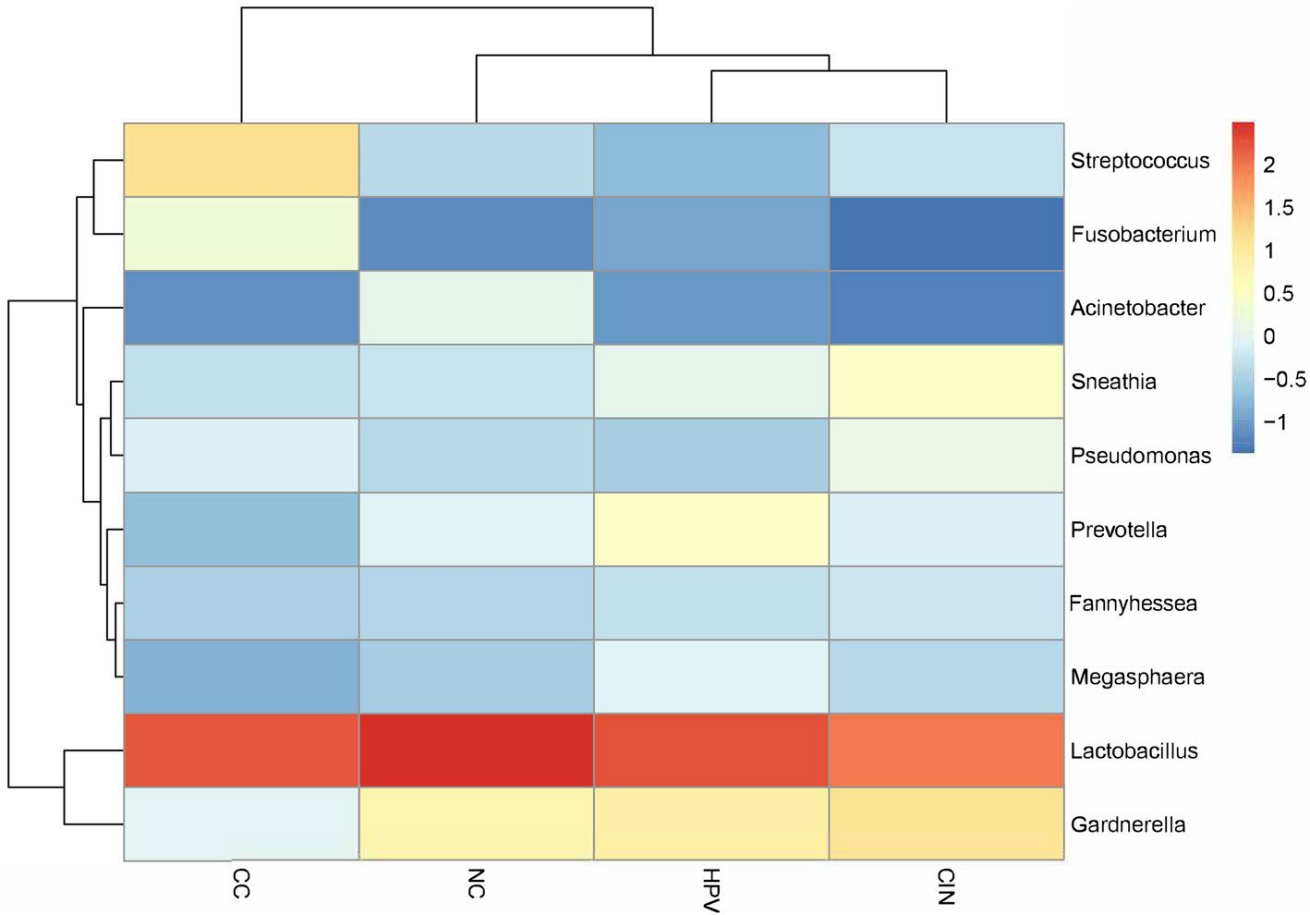
Heatmap plot showing the top 10 most abundant bacterial communities in each group at the genus level. The abundance was log-transformed to reduce the skewness of the data.

**Figure 3:**
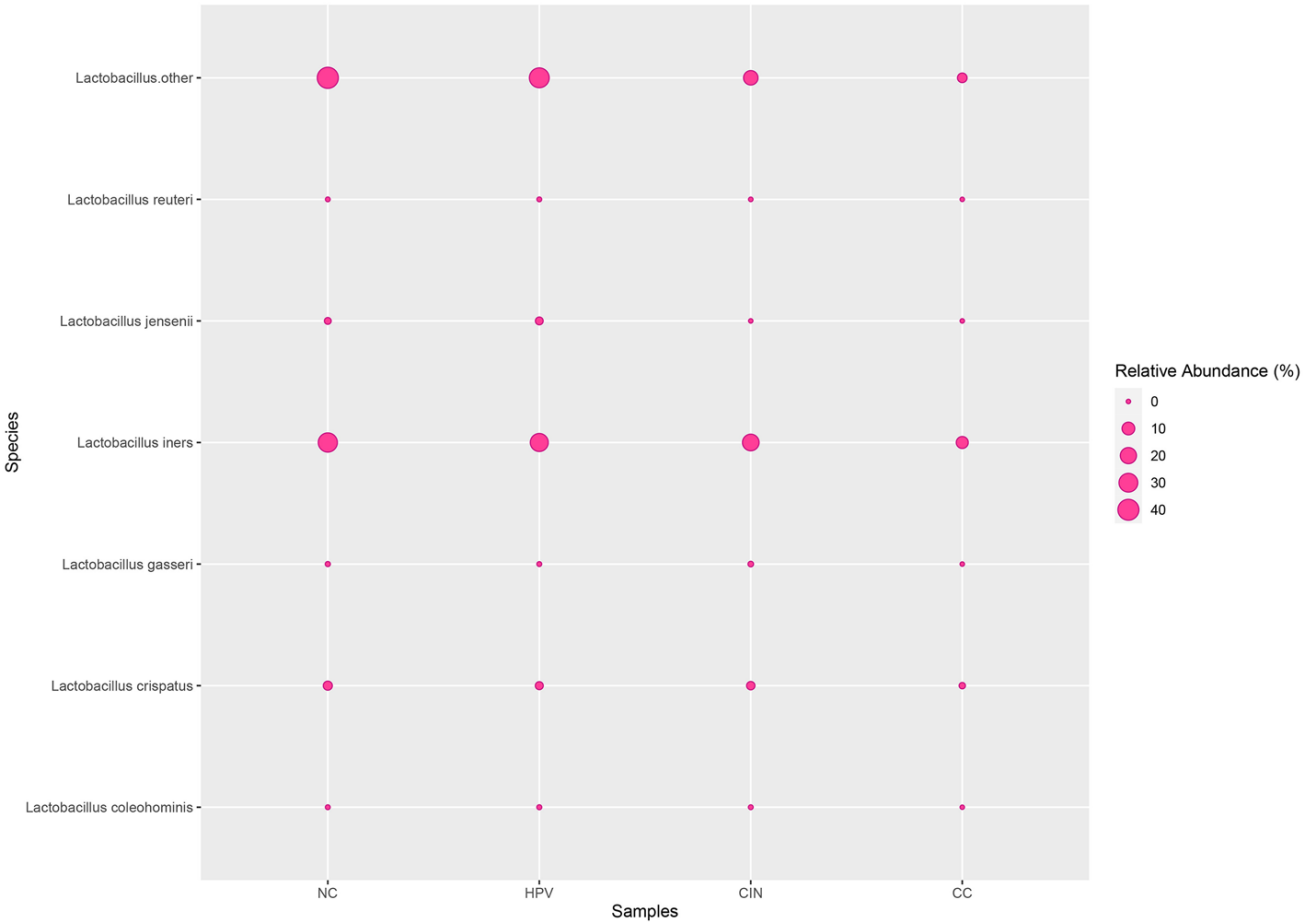
Bubble plot showing relative abundance of *Lactobacillus* species across stage groups.

**Figure 4:**
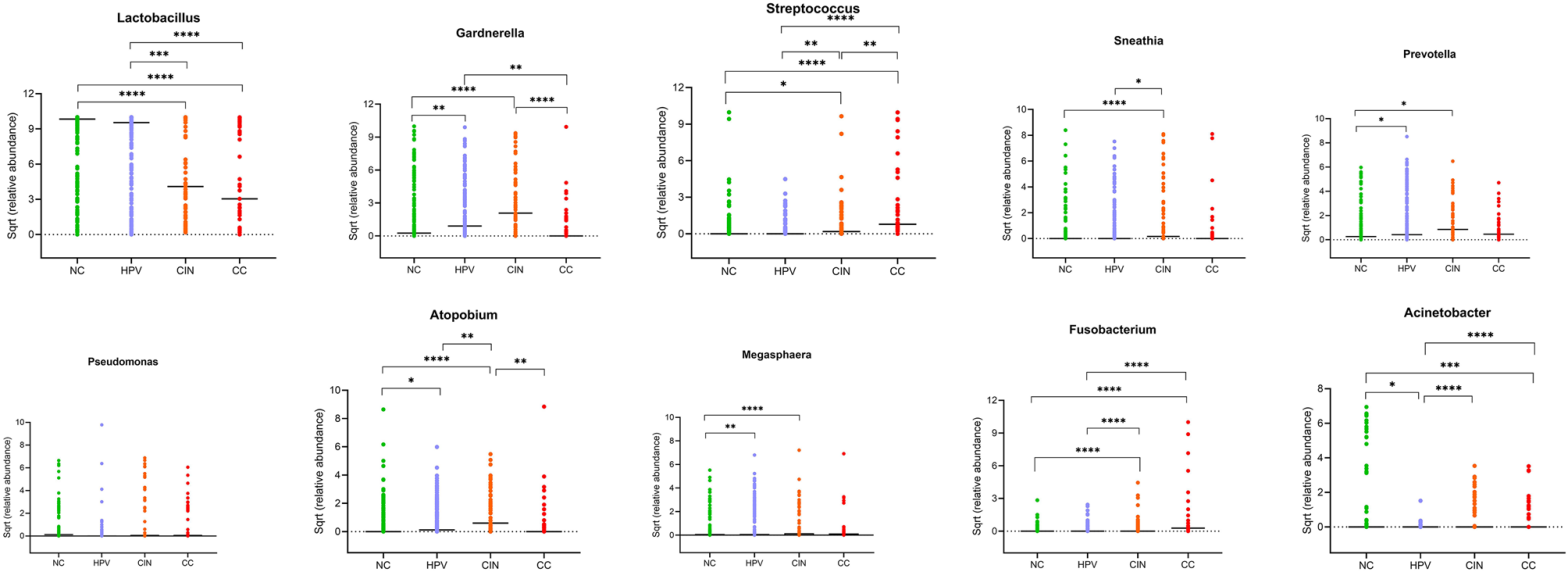
Comparison of top 10 abundant bacterial genera across disease progression stages. Multiple groups comparisons were peformed using Kruskal–Wallis test. *p < 0.05; **p < 0.01; ***p < 0.001; ****p < 0.0001.

The present study investigated the comprehensive microbial compositions during the course of cervical cancer. The cervical microbiota was shown to be predominantly composed of five bacterial phyla at the phylum level, namely *Firmicutes*, *Actinobacteria*, *Proteobacteria*, *Bacteroidetes*, and *Fusobacteria*. There were 45 genera identified as core microbiota when combining all datasets. Among them, *Lactobacillus*, *Gardnerella*, *Streptococcus*, *Sneathia*, *Prevotella*, *Pseudomonas*, *Fannyhessea*, *Megasphaera*, *Fusobacterium* and *Acinetobacter* were the most prevalent and abundant genera across the datasets. Hierarchical clustering analysis showed that the HPV group and CIN group shared a similar distribution of the top 10 genera (Fig. 2). For *Lactobacillus*, the most common encountered species was *L. iners* (Fig. 3). Moreover, *Lactobacillus* was significantly lower in CIN and CC groups when compared to NC and HPV groups (*p* < 0.001). While, *Streptococcus* and *Fusobacterium* were significantly higher in CIN and CC groups when compared to NC and HPV groups (*p* < 0.05) (Fig. 4).

### Differential taxa across different disease stages

**Figure 5:**
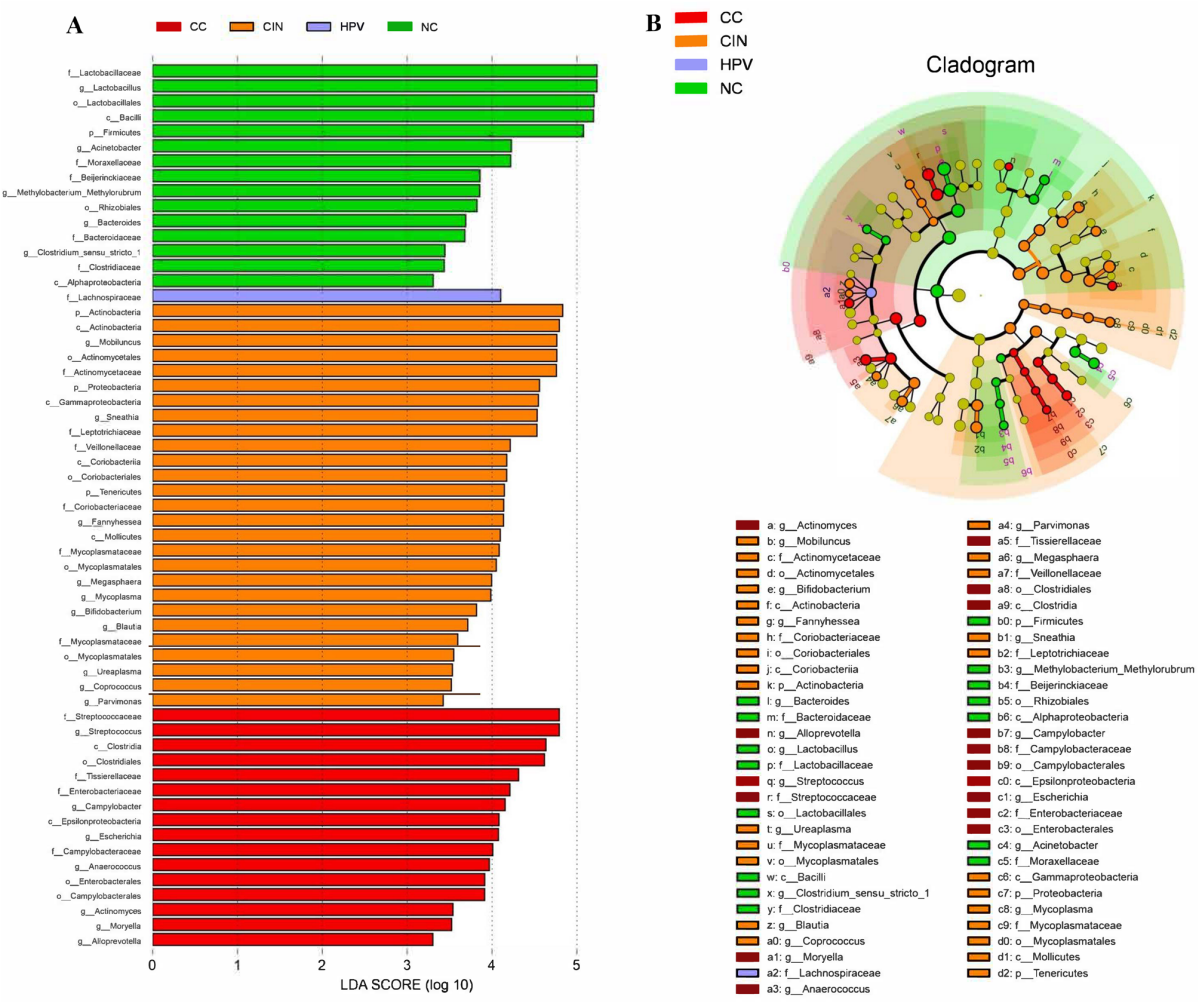
Bacterial taxa differences among the four groups using Wekemo Bioincloud (https://bioincloud.tech/). (**A**) Linear discriminant analysis (LDA) effect size (LEfSe) analysis on selected core taxa among the four groups. Only lineages with LDA values > 3 are displayed. (**B**) Cladogram showing the phylogenetic distribution of lineages associated with the four groups.

LEfSe was performed to identify the specific taxa with significantly higher abundance among the four groups. As shown in Fig. 5, a total of 59 clades were screened out with a LDA threshold score of 3.0. Class *Bacilli* (including *Lactobacillus* genus) was enriched in the NC group. Genus *Acinetobacter* and *Bacteroides* were also enriched in the NC than in other groups. The CIN group was more highly colonized by class *Actinobacteria* and *Gammaproteobacteria*. As for the CC group community, class *Clostridia* and *Epsilonproteobacteria* had significantly higher relative abundances.

### Bacterial biomarkers for distinguishing CC from NC

**Figure 6:**
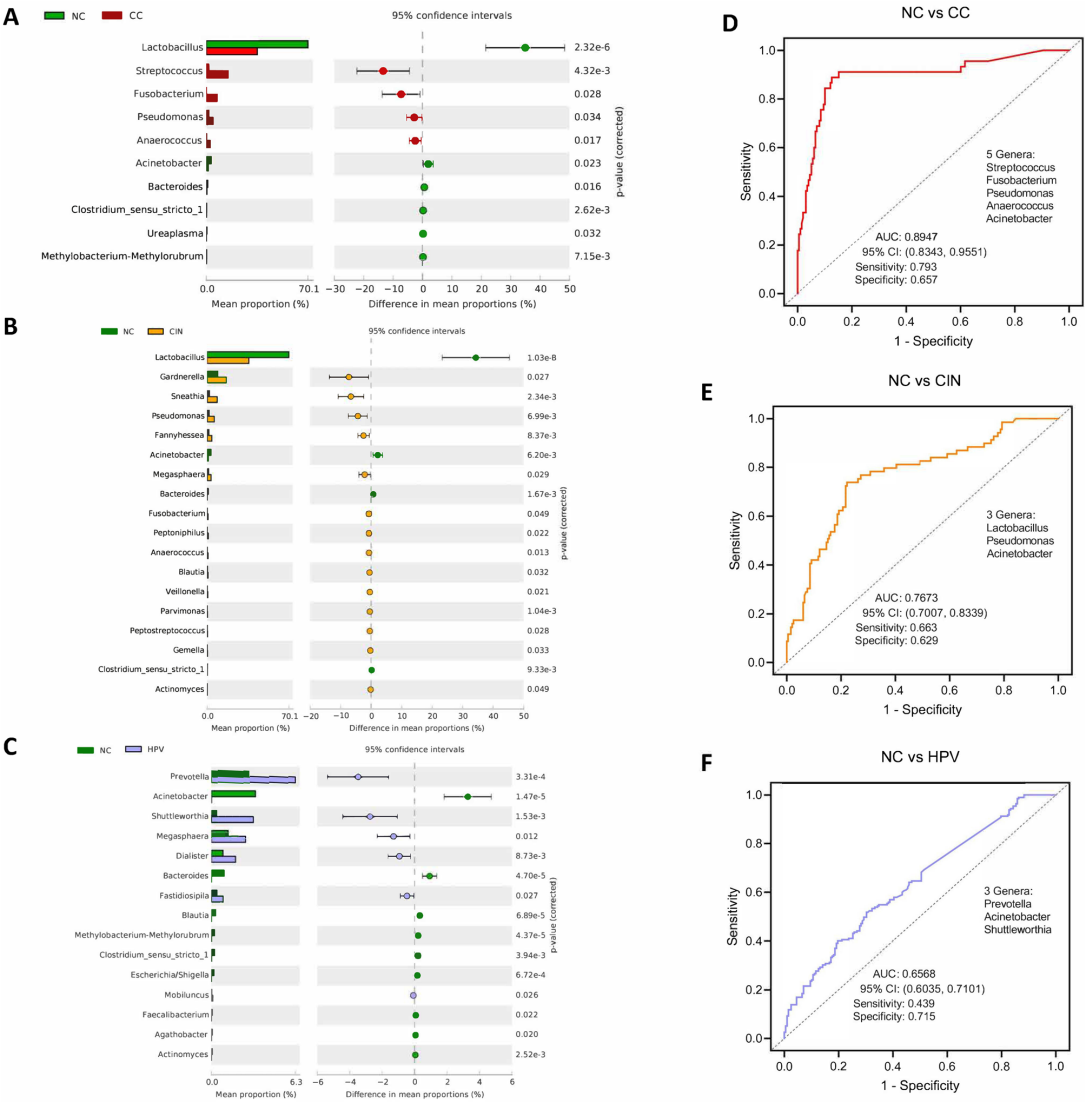
Differentially taxa between CC, CIN, HPV and NC and the diagnostic genera markers. (**A**–**C**) The significantly altered genera as revealed by the extended error bar method using the Wilcoxon rank-sum test. (**D**–**F**) Receiver operating characteristic (ROC) analysis for the identified genera markers with logistic regression model discriminating CC, CIN, and HPV from NC.

Among the ten bacterial genera that significantly differed between the two stages, four genera including *Streptococcus*, *Fusobacterium*, *Pseudomonas* and *Anaerococcus*, were found to be abundant in the CC stage compared to the NC stage. On the other hand, six were depleted including *Lactobacillus*, *Acinetobacter* and *Bacteroides* (Fig. 6A). We assessed the significantly altered bacterial genera for their potential as diagnostic biomarkers for discriminating CC from NC. Four CC-enriched and one CC-depleted were identified as potential biomarkers using the backward stepwise selection algorithm. A logistic regression model was built based on the five biomarkers. To evaluate the performance of the model, ROC analysis was conducted, yielding an area under the curve (AUC) of 0.8947 (95%CI: 0.8343, 0.9551) (Fig. 6D).

### Potential biomarkers to classify other disease stages

Microbial biomarkers were identified with the purpose of developing invasive diagnostic procedures for distinguishing between CIN and NC, HPV infection and NC, CIN and HPV infection, CC and HPV infection, and CC and CIN. The microbial taxa that exhibited significant differences between CIN and NC samples consisted of 14 genera that were enriched in CIN samples and 4 genera that were deficient in CIN samples (Fig. 6B). The logistic regression model, which utilised three genera, effectively differentiated between CIN and NC. It achieved an AUC of 0.7673 (95% Confidence Interval [CI]: 0.7007, 0.8339) (Fig. 6E). Nevertheless, the model’s ability to differentiate between HPV and NC was deemed inadequate, as indicated by the AUC of 0.6568 (95% confidence interval: 0.6035, 0.7101) (Fig. 6C, F). Similarly, the performance of the models for distinguishing the CIN from HPV was good (AUC: 0.7669, 95%CI: 0.6919, 0.8418) (Figure S1A). The taxa were significantly changed between CC and HPV including 9 CC-enriched and 5 CC-depleted genera (Figure S1B). Five genera were capable of differentiating samples between CC and HPV (AUC: 0.8836, 95%CI: 0.8217, 0.9455) (Figure S1E). For CC vs CIN, 2 CC-enriched genera and 1 CC-depleted genus were significantly altered (Figure S1C), these genera were capable of discriminating samples between CC and CIN by achieving an AUC of 0.7353 (95%CI: 0.6422, 0.8284) (Figure S1F).

### Correlation network analysis

**Figure 7:**
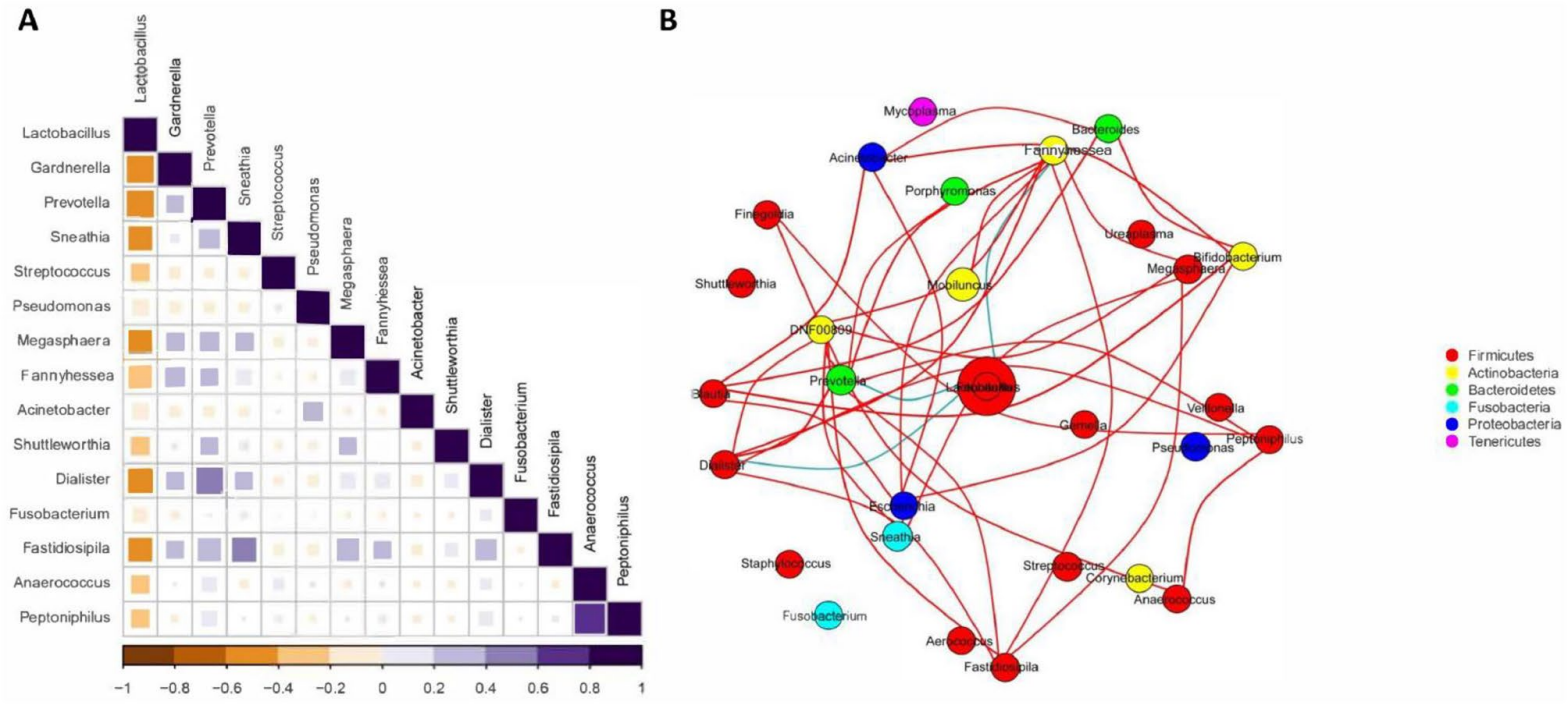
(**A**) Correlation analysis of the top 15 dominant genera with spearman^,^s rank correlation test method using R software (version 4.1.2). (**B**) Correlation network of the core genera in the combined dataset using Wekemo Bioincloud (https://bioincloud.tech/). The blue line indicates a negative correlation (r < − 0.6), and the red line indicates a positive correlation (r > 0.6). Each circle represents a core bacterial taxa, and the colour of the circle represents the phylum to which it belongs.

To further our comprehension of the potential interaction among core taxa, we performed co-occurrence network analysis in the datasets. Significant correlations were found in 35 genera pairs (r > 0.6 or r < − 0.6, *p* < 0.05) (Table S1). There were three genera pair showing negative correlations. *Lactobacillus* exhibited a negative correlation compared with all other dominant genera (Fig. 7A). The most strongest negative correlation was found between *Lactobacillus* and *Prevotella*. In addition, *Lactobacillus* also had negative correlations with *Fannyhessea* and *Dialister*. Positive co-occurrence correlations occurred in the genera of *Prevotella*, *Sneathia*, *Megasphaera*, *Fannyhessea, Acinetobacter, Dialister, Anaerococcus* and *Bifidobacterium* (Fig. 7B). These microorganisms may play a crucial role in the network.

## Discussion

Cervical cancer is a multifactorial disease involving the interactions among host, microbial and environmental factors. Despite HPV being demonstrated as the main well-established risk factor, cervicovaginal microbiome dysbiosis has emerged as a key risk factor in inflammation^50^, HPV acquisition, persistence and cervical carcinogenesis^19^. We integrated six datasets to present a cervical-related microbial landscape along the different stages, and explored the potential bacterial taxa as biomarkers to monitor cervical carcinogensis.

As an important component of the cervicovaginal self-purification function and biological barrier, the resident microbiota and opportunistic pathogens of the cervix maintain the microecological balance. The dominant bacteria *Lactobacillus* can decompose cervical epithelial glycogen to produce lactic acid, maintain a weakly acidic environment, which can inhibit the proliferation of pathogens^51^. In addition, they can enhance anti-infection ability by producing various metabolites or stimulating immune cells to produce various cytokines^52^. Following imbalance of this defense system, it may induce histological alterations of the vaginal mucosa and the cervical epithelium, thereby exerting a selective pressure on the microbiota^53^. Some cervicovaginal taxa, such as *Gardnerella*, *Fusobacteria*, *Dialister* and *Prevotella*, as well as a decrease in the proportion of *Lactobacillus* spp. have been linked to dysbiosis that would generate an unstable microenvironment, which in turn may affect key risk factor in cervical cancer^54,55^. Acid-producing *Lactobacillus* dysbiosis are responsible for increasing the levels of mucin-degrading enzymes, which may affect the mucous layer stability of the cervicovaginal epithelium^56^.

We observed no significant difference between HPV and NC group in bacterial richness and diversity, which is consistent with several previous studies^15,57,58^. Although a few studies results showed that HPV infection can increase bacterial diversity^13,43,59^. The prevailing bacterial taxa that exhibited enrichment in women with HPV infection were *Prevotella*, *Megasphaera*, *Shuttleworthia*, and *Dialister*. This finding aligns with prior investigations conducted in this field^60,61^. *Prevotella* has been considered to play an important role in HPV infection and persistence^43^. This bacterium was indicated as a major modulator of host inflammatory responses in the female genital tract by increasing the number of cytokines in cervicovaginal fluid^62^. Moreover, *Prevotella* can secrete proteases to degrade host antibodies, and transfer ammonia to *Gardnerella*, resulting reduction in host mucosal immunity^63^. Lebeau et al.^64^ found that HPV infection alters vaginal microbiome through down-regulating host mucosal innate peptides used by *Lactobacilli* as amino acid sources. However, several studies have shown that HPV does not necessarily induce significant changes in the cervicovaginal microbial communities^15,65^. Borgogna et al.^31^ found that the vaginal metabolome of HPV-positive women differed from normal individuals in terms of several metabolites, including biogenic amines, glutathione and lipid-related metabolites.

Higher richness and diversity were observed in CIN and CC groups when compared to NC group. *Gardnerella*, one of the most frequently reported in CIN studies, was enriched in CIN group compared with NC, as well as other undesirable genera,like *Sneathia*, *Pseudomonas*, *Fannyhessea* and *Megasphaera*. A longitudinal cohort study revealed a positive association between *Gardnerella* and CIN progression caused by elevated microbial diversity^66^. Previous studies have posited that there may be an increased risk of CIN associated with the enrichment of *Gardnerella vaginalis* (*G. vaginalis*) and *Fannyhessea vaginae*^67^. *G. vaginalis* is classified as a facultative anaerobe, capable of adhering to the vaginal epithelium. This adherence serves as a framework for the production of biofilms and promotes the proliferation of microorganisms^68,69^. It was associated with higher levels of inflammatory cytokines^70^. It has been reported that *Fannyhessea vaginae* can activate the proinflammatory transcription factor NF-*k*B in the cervicovaginal epithelial cells, triggering abundant inflammation and innate immune responses^71,72^.

In relation to CC, it has been observed that some bacterial taxa, namely *Fusobacterium*, *Pseudomonas*, and *Anaerococcus*, exhibit not only higher abundance in women with precancerous lesions but also in women with cervical cancer. Other identified taxon in this meta-analysis to be associated with cervical cancer included *Streptococcus*. Previously, species of *Streptococcus* have been reported that likely involve the activation of multiple inflammatory cytokines and may affect human vaginal and cervical epithelial cells. Soares et al.^73^ revealed that *Streptococcus* possesses metallopeptidases which could help them invade tissue or cause bacterial transmission. *Fusobacterium* species have attracted widespread attention due to their pro-inflammatory properties^74,75^. *Fusobacterium nucleatum* has been shown to potentiate intestinal tumorigenesis and modulate the tumor-immune microenvironment, indicating potential as a diagnostic biomarker for colorectal cancer^76,77^. Besides, *Fusobacterium nucleatum* also has been studied as a possible diagnostic biomarker of CC as it is positively correlated with tumor differentiation^78^. *Pseudomonas* has long been considered to be an opportunistic pathogen in vaginal inflammation, the human urogenital system. They can disrupt the mucosal defense against extracellular pathogens by secreting protease IV and inactivating interleukin 22^79^. It has been indicated that *Pseudomonas aeruginosa* has a potential role in the development of cervical cancer by promoting the expression of integrins in cervical cancer cells^80^.

In this meta-analysis, we observed distinct distributions of the CC group compared with other sample groups. The most relevant genera in each disease stage were revealed by our study, which allowed us to discover robust diagnostic biomarkers. Finally, five common genera including *Streptococcus*, *Fusobacterium*, *Pseudomonas*, *Anaerococcus* and *Acinetobacter* were identified as the most important features for distinguishing CC from the normal population (AUC > 0.8), indicating their possible role in cervical carcinogenesis as well as their clinical applications. Additionally, we also identified the depletion of potentially beneficial microorganisms, such as lactic acid-producing *Lactobacillus*. It has been reported that *L. iners* can create a protective micro-ecological environment by regulating the core fucosylation of the vaginal epithelial cell against CC^44^. Moreover, we identified additional biomarkers to discriminate CC from CIN (AUC > 0.7) and HPV (AUC > 0.8).

Profiling cervicovaginal microbial communities using 16S rRNA genes is a straightforward and cost-effective method, and is generally cheaper than shotgun metagenomic sequencing. Nevertheless, shotgun metagenomics and circularizing probes-based RNA (ciRNA) sequencing targeted approaches with species-level resolution can provide in-depth insights into cervicovaginal microbiome. A growing number studies have employed shotgun sequencing of the vaginal metagenome^41,81–84^, metatranscriptome^85,86^ and ciRNA^87,88^. Liu et al.^84^ have observed a total of 111 species in vaginal microbiome of healthy Chinese women, including all dominant vaginal *Lactobacillus* species, such as *L. iners*, *L. crispatus*, *L. gasseri*, and *L. jensenii*. It has been reported that *L. crispatus*, *L. iners* and *G. vaginalis* were the top three species in both HPV16-positive and control groups^83^. A previous study by Macklaim et al.^85^ focused on the transcriptional activity of *L. iners* in four reproductive age women and found variation in the species, transcriptional activity. Communities dominated by *L. crispatus* were found to exhibit higher expression of phosphate and phosphonate transporters^86^.

The meta-analysis conducted for CIN and CC in our study was constrained by limitations in the study number and sample size. Additionally, the study lacks comprehensive demographic and clinical data. Therefore, we appeal researchers to accurately and comprehensively disseminate their sequencing data and accompanying metadata. Furthermore, the division of CIN samples into low-grade squamous intraepithelial lesions (LSIL) and high-grade squamous intraepithelial lesions (HSIL) was not feasible due to the limited sample size. Moreover, the fact that LSIL are considered non-progressive lesions, which could affect the analyses in the study.

## Conclusions

In summary, the present study conducted a comprehensive analysis of several cervical 16S rRNA gene sequencing datasets in a standardised manner, leading to the identification of distinct microbial characteristics in the cervical region throughout different stages of disease progression. The findings of our investigation suggest a potential correlation between the presence of certain microorganisms and the development of cervical cancer, which provides great clinical significance and feasibility for the development of noninvasive screening or diagnosis methods for cervical cancer.

### Supplementary Information

Supplementary Information.

## Data Availability

The original contributions presented in the study are included inthe article/Supplementary Material. Further inquiries can be directed to the corresponding author.
