# Supplementary material for: Leveraging existing 16S rRNA gene surveys to decipher microbial signatures and dysbiosis in cervical carcinogenesis
Source: Sci Rep. 2024 May 21;14:11532. doi: 10.1038/s41598-024-62531-z (PMC11109339; doi:10.1038/s41598-024-62531-z)
Supplement: Supplementary file 1 — Supplementary Information. [file 41598_2024_62531_MOESM1_ESM.docx]

**Leveraging existing 16S rRNA gene surveys to decipher microbial signatures and dysbiosis in cervical carcinogenesis**

Xiaoxiao Li^1#^, Fenfen Xiang^1#^, Tong Liu^2^, Zixi Chen^1^, Mengzhe Zhang^1^, Jinpeng Li^1^, Xiangdong Kang^1*^, Rong Wu^1*^

^1^Laboratory Medicine, Putuo Hospital, Shanghai University of Traditional Chinese Medicine, Shanghai, China

^2^Department of Molecular Science, Uppsala Biocenter, Swedish University of Agricultural Science, Uppsala, Sweden

**^#^**These authors contributed equally to this work

***Corresponding authors:** Rong Wu and Xiangdong Kang, Department of Laboratory Medicine, Putuo Hospital, Shanghai University of Traditional Chinese Medicine, Shanghai, China.

164 Lanxi Road, Shanghai 200062, P. R. China

E-mail: rong701@126.com (R. W.) and xd_kang@163.com (X. K.) ; Telephone: 021-51322043

Table S1 Genera pairs detected by co-occurrence network analysis ( r > 0.6 or r < -0.6, p < 0.05).

| Source | Target | Correlation | p-value | Correlation_type |
| --- | --- | --- | --- | --- |
| Lactobacillus | Prevotella | -0.66427 | 7.93E-66 | negative |
| Lactobacillus | Fannyhessea | -0.63437 | 1.92E-58 | negative |
| Lactobacillus | Dialister | -0.61403 | 7.28E-54 | negative |
| Mobiluncus | Fannyhessea | 0.650007 | 3.36E-62 | positive |
| Prevotella | Fannyhessea | 0.684326 | 2.78E-71 | positive |
| Prevotella | Dialister | 0.766901 | 2.45E-99 | positive |
| Prevotella | Fastidiosipila | 0.619687 | 4.20E-55 | positive |
| Prevotella | Peptoniphilus | 0.648569 | 7.61E-62 | positive |
| Prevotella | Porphyromonas | 0.618394 | 8.11E-55 | positive |
| Prevotella | DNF00809 | 0.681702 | 1.52E-70 | positive |
| Sneathia | Megasphaera | 0.620065 | 3.47E-55 | positive |
| Sneathia | Fannyhessea | 0.605501 | 4.84E-52 | positive |
| Sneathia | DNF00809 | 0.620417 | 2.90E-55 | positive |
| Megasphaera | Fannyhessea | 0.610674 | 3.86E-53 | positive |
| Megasphaera | Fastidiosipila | 0.621153 | 1.99E-55 | positive |
| Megasphaera | DNF00809 | 0.644855 | 6.15E-61 | positive |
| Fannyhessea | Dialister | 0.706283 | 8.82E-78 | positive |
| Fannyhessea | Fastidiosipila | 0.610998 | 3.29E-53 | positive |
| Fannyhessea | DNF00809 | 0.681585 | 1.64E-70 | positive |
| Acinetobacter | Bifidobacterium | 0.701116 | 3.38E-76 | positive |
| Acinetobacter | Bacteroides | 0.600229 | 6.08E-51 | positive |
| Acinetobacter | Blautia | 0.713679 | 4.15E-80 | positive |
| Acinetobacter | Escherichia | 0.635105 | 1.30E-58 | positive |
| Dialister | Fastidiosipila | 0.607011 | 2.32E-52 | positive |
| Dialister | Peptoniphilus | 0.610655 | 3.90E-53 | positive |
| Dialister | DNF00809 | 0.636689 | 5.52E-59 | positive |
| Fastidiosipila | DNF00809 | 0.678315 | 1.33E-69 | positive |
| Anaerococcus | Peptoniphilus | 0.693454 | 6.51E-74 | positive |
| Anaerococcus | Finegoldia | 0.635846 | 8.71E-59 | positive |
| Peptoniphilus | Finegoldia | 0.601316 | 3.62E-51 | positive |
| Bifidobacterium | Bacteroides | 0.683292 | 5.45E-71 | positive |
| Bifidobacterium | Blautia | 0.786633 | 7.89E-108 | positive |
| Bifidobacterium | Escherichia | 0.645319 | 4.75E-61 | positive |
| Bacteroides | Blautia | 0.708358 | 2.00E-78 | positive |
| Blautia | Escherichia | 0.628991 | 3.38E-57 | positive |


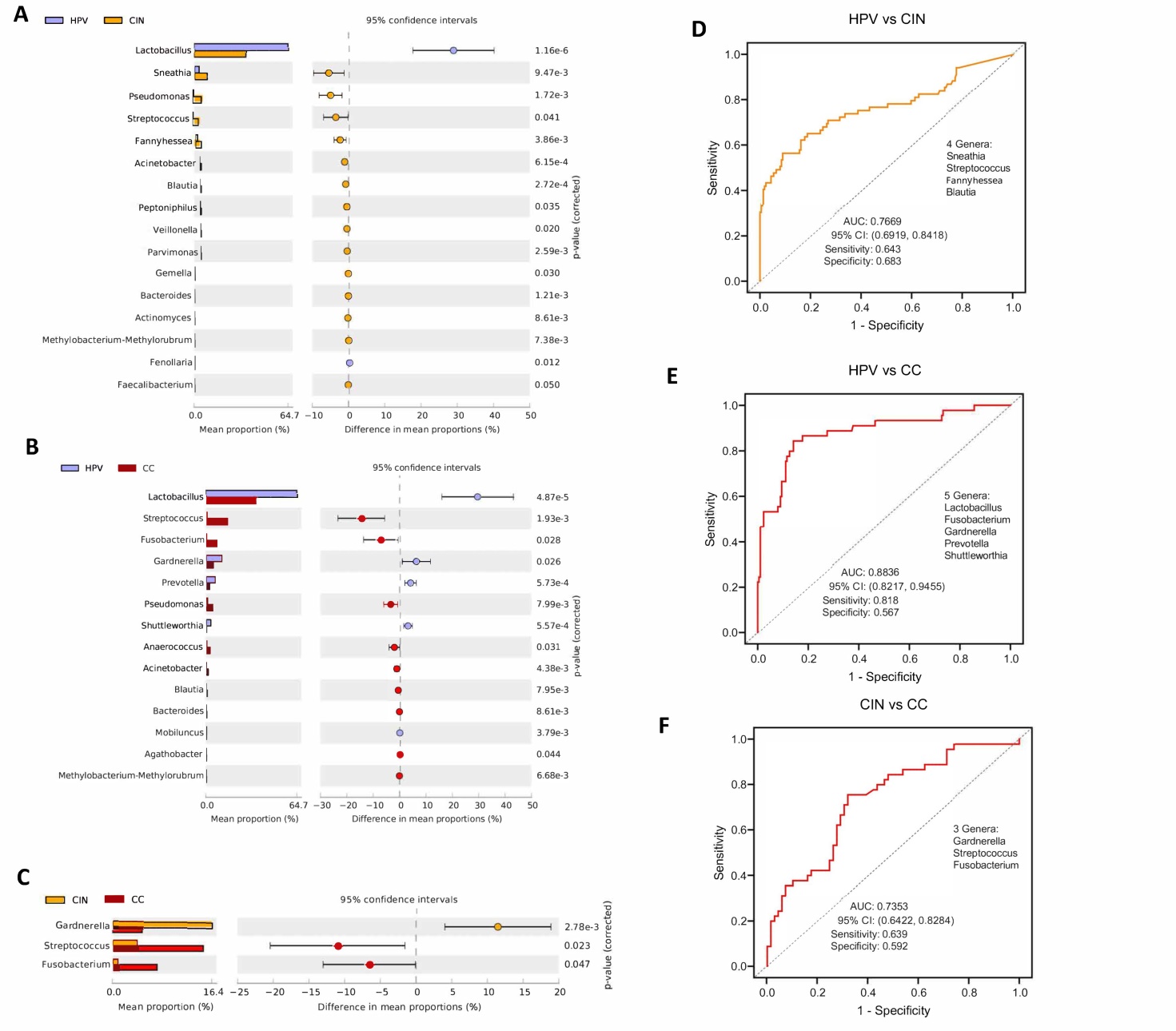


**Figure S1** Differentially taxa between CC, CIN and HPV, CIN and CC, and the diagnostic genera markers. (A-C) The significantly altered genera as revealed by the extended error bar method using Wilcoxon rank-sum test . (D-F) Receiver operating characteristic (ROC) analysis for the identified genera markers with logistic regression model discriminating CC, CIN from HPV, CIN from CC.
